# Major adverse cardiac events and mortality associated with ADHD and its treatment in adults with type 2 diabetes: a multi-national cohort study

**DOI:** 10.1186/s12916-026-05246-4

**Published:** 2026-09-26

**Authors:** Zihan Dong, Yiling Zhou, Honghui Yao, Shengxin Liu, Aske Astrup, Anders Engeland, Le Gao, Malcolm B. Gillies, Astrid Lunde, Yanli Zhang-James, Masako Araki, Isabell Brikell, Agnieszka Butwicka, Søren Dalsgaard, Brian M. D’Onofrio, Stephen V. Faraone, Soffia Gudbjornsdottir, Jan Haavik, Ralf Kuja-Halkola, Kari Klungsøyr, Henrik Larsson, Kenneth K. C. Man, Sallie-Anne Pearson, Dina Sarsembayeva, Melissa Vos, Ian C. K. Wong, Helga Zoega, Harold Snieder, Catharina A. Hartman, Zheng Chang, Ebba Du Rietz

**Affiliations:** 1 Department of Medical Epidemiology and Biostatistics, Karolinska Institutet, Stockholm, Sweden; 2 Department of Epidemiology, University of Groningen, University Medical Center Groningen, Groningen, Netherlands; 3 Department of Psychiatry, The Second Xiangya Hospital, Central South University, Changsha, Hunan China; 4 National Centre for Register-based Research, Aarhus University, Aarhus, Denmark; 5 Centre for Integrated Register-based Research, Aarhus University, Aarhus, Denmark; 6 Department of Chronic Diseases Norwegian Institute of Public Health, Bergen, Norway; 7 Department of Global Public Health and Primary Care, University of Bergen, Bergen, Norway; 8 Department of Pharmacy Administration, School of Pharmacy, Xi’an Jiaotong University, Shaanxi, China; 9 Centre for Safe Medication Practice and Research, Department of Pharmacology and Pharmacy, Li Ka Shing Faculty of Medicine, The University of Hong Kong, Hong Kong Special Administrative Region, Hong Kong, China; 10 School of Population Health, Faculty of Medicine and Health, University of New South Wales, Sydney, NSW Australia; 11 Department of Psychiatry and Behavioral Sciences, Norton College of Medicine, SUNY Upstate Medical University, Syracuse, NY USA; 12 Department of Biomedicine, Aarhus University, Aarhus, Denmark; 13 Division of Mental Health Services, Akershus University Hospital, Lørenskog, Norway; 14 Institute of Clinical Medicine, University of Oslo, Oslo, Norway; 15 Department of Medical Sciences, Child and Adolescent Psychiatry, Uppsala University, Uppsala, Sweden; 16 Department of Biostatistics and Translational Medicine, Medical University of Lodz, Lodz, Poland; 17 Child and Adolescent Mental Health Center, Copenhagen University Hospital – Mental Health Services CPH, Copenhagen, Denmark; 18 Department of Clinical Medicine, University of Copenhagen, Copenhagen, Denmark; 19 Department of Psychological and Brain Sciences, Indiana University, Bloomington, USA; 20 Department of Molecular and Clinical Medicine, Sahlgrenska Academy, University of Gothenburg, Gothenburg, Sweden; 21 Division of Psychiatry, Haukeland University Hospital, Bergen, Norway; 22 Department of Biomedicine, Faculty of Medicine, University of Bergen, Bergen, Norway; 23 Department of Health Promotion, Norwegian Institute of Public Health, Bergen, Norway; 24 School of Medical Sciences, Örebro University, Örebro, Sweden; 25 Research Department of Practice and Policy, School of Pharmacy, University College London, London, UK; 26 Centre for Medicines Optimisation Research and Education, University College London Hospitals NHS Foundation Trust, London, UK; 27 Centre for Safe Medication Practice and Research, Department of Pharmacology and Pharmacy, Li Ka Shing Faculty of Medicine, University of Hong Kong, Hong Kong, China; 28 Department of Psychiatry, Interdisciplinary Center of Psychopathology and Emotion Regulation, University of Groningen, University Medical Center Groningen, Groningen, Netherlands; 29 School of Pharmacy, Medical Sciences Division, Macau University of Science and Technology, Macao Special Administrative Region, Macao, China; 30 Aston Pharmacy School, Aston University, Birmingham, UK; 31 Centre of Public Health Sciences, Faculty of Medicine, University of Iceland, Reykjavik, Iceland

**Keywords:** ADHD, ADHD medications, Type 2 diabetes, Cardiovascular diseases, Major adverse cardiac events, Mortality, Multi-country study

## Abstract

**Background:**

Attention-deficit/hyperactivity disorder (ADHD) has been linked to elevated risk of type 2 diabetes (T2D). However, the impact of ADHD and ADHD medication use on prognosis and health outcomes in T2D remains unclear. This study investigates the associations of (1) ADHD and (2) ADHD medication use with rates of major adverse cardiac events (MACE) and all-cause mortality after a T2D diagnosis.

**Methods:**

This multinational cohort study used population-based data from Australia, Denmark, the Netherlands, Norway, Sweden, the United Kingdom, and the United States. Using a standardized analytical protocol, we identified adults with a first recorded T2D diagnosis during 2010-2020. Exposures were (1) ADHD, defined by a recorded ADHD diagnosis or ADHD medication dispensation, and (2) among adults with ADHD, the use of ADHD medication, both analysed as time-varying exposures. Main outcomes were MACE (acute myocardial infarction, stroke, or cardiovascular disease-related death) and all-cause mortality after T2D diagnosis. Cause-specific Cox models estimated hazard ratios (HRs) with 95% confidence intervals (CIs); results were pooled using random-effects meta-analyses and stratified by age and sex.

**Results:**

The cohort included 1,929,027 adults with T2D (909,176 [47.1%] males). In pooled analyses, adults with T2D and ADHD had a higher rate of all-cause mortality (HR, 2.42; 95% CI, 1.72-3.41), but not MACE (HR, 1.04; 95% CI, 0.81-1.35), after a T2D diagnosis compared with those without ADHD. Among adults with T2D and ADHD, ADHD medication use was associated with higher rate of MACE in those diagnosed with T2D before age 45 years (HR, 1.58; 95% CI, 1.18-2.10). These associations remained consistent, after adjusting for sex, age, calendar year at T2D diagnosis, psychiatric history, other psychotropic medication use, and cardiometabolic medication use.

**Conclusions:**

Adults with T2D and co-occurring ADHD were found to have increased risk of all-cause mortality after T2D diagnosis, while ADHD was not consistently associated with MACE across countries. ADHD medication use was linked to higher rate of MACE in adults with early-onset T2D. These findings suggest the potential need for integrated somatic-psychiatric care and careful consideration of ADHD medication use that may help reduce MACE and mortality, particularly in younger-onset T2D.

## Background

Attention-deficit/hyperactivity disorder (ADHD) is a neurodevelopmental condition characterized by persistent inattention, hyperactivity, and impulsivity that impair daily functioning. ADHD is diagnosed in around 5% of school-aged children, and at least two-thirds continue to have impairing symptoms in adulthood [1]. Use of ADHD medications, both stimulants and non-stimulants, has risen markedly in the past decades [2] and a multi-national study found that 30-60% of individuals with ADHD remain on treatment for up to five years [3].

Growing evidence from large observational studies suggests that ADHD is associated with up to a two-fold increased risk of type 2 diabetes (T2D), a common metabolic disorder characterized by insulin resistance and persistent hyperglycemia [4–7]. T2D is a well-established risk factor for both cardiovascular diseases (CVDs) [8], and mortality [9], particularly major adverse cardiac events (MACE), including myocardial infarction, stroke, and cardiovascular death [10]. These outcomes in T2D are often linked to cardiometabolic risk factors such as obesity, dyslipidaemia, and hypertension [8].

Large population-based studies have also found that ADHD is linked to CVDs [11] and all-cause mortality [12]. On the other hand, the impact of ADHD medications on long-term health remains uncertain. Intriguingly, studies have shown that long-term use may increase CVD risk [13,14], but also that initiating medication can reduce unnatural-cause mortality related to unintentional injuries, suicide, and accidental poisonings [15].

Effective management of T2D requires adherent medication use, lifestyle modification, regular blood glucose monitoring and routine medical follow-ups [16]. These demands involve organization, planning, and sustained attention [17], which are particularly challenging for individuals with ADHD. Nonetheless, evidence on the impact of ADHD on T2D outcomes remains limited and inconsistent. One small study (n = 315) reported no association between ADHD-like symptoms and haemoglobin A1c (HbA1c), lipid profile or estimated glomerular filtration rate (eGFR) in individuals with T2D [18], whereas another larger study (*n* = 3,164) found that individuals with co-occurring T2D and ADHD showed poorer glycaemic control [19].

Given that individuals with T2D have a higher risk of CVD and all-cause mortality, and that ADHD is independently associated with similar adverse outcomes and may interfere with diabetes self-management, it is important to determine the extent to which ADHD further exacerbates these risks in individuals with T2D. Understanding this relationship could inform integrated and targeted care strategies to improve outcomes in this population. In this study, we investigated the extent to which adults with newly diagnosed T2D, with and without ADHD, differ in the incidence of MACE and all-cause mortality using national administrative or registry data from seven countries. We also investigated the extent to which ADHD medication use is associated with the rate of MACE and all-cause mortality in people with co-occurring T2D and ADHD.

## Methods

### Data sources

We used real-world individual-level data from seven countries (Australia, Denmark, the Netherlands, Norway, Sweden, the United Kingdom [UK], and the United States [US]), linked with health records and medication dispensations (Additional File 1: Table S1 for details). For most countries, data on diagnosis and medication prescription or dispensation (using term “dispensation” hereafter) were available from January 1, 2009 to December 31, 2020 **(s**ee exceptions in Additional File 1: Table S1**)**.

Ethical approval was obtained for the use of each data source. Informed consent was not required as the study relied on registry and administrative data **(**Additional File 1: sMethods**)**. We adhered to Strengthening the Reporting of Observational Studies in Epidemiology (STROBE) guidelines for cohort studies [20].

### Study population and design

Eligible adults were aged at least 18 years and had the first record of a T2D diagnosis (International Statistical Classification of Diseases, Tenth Revision [ICD-10]-code E11) or prescription of blood glucose-lowering medication, excluding insulins (Anatomical Therapeutic Chemical [ATC]-code A10B) between 2010 and 2020. We defined adults with newly diagnosed T2D using a wash-out period of ≥12-months without any records of T2D diagnosis or blood glucose-lowering medication dispensation. We excluded adults with missing data on sex or birth date, or had emigrated, died, had prior records of MACE (Additional File 1: Table S2), or had type 1 diabetes (T1D) (ICD-10-code E10; ICD-8/9-code 250 before 18 years, or were only prescribed insulin: ATC-code A10A) before or at T2D diagnosis. Exclusions depended on the available data in each country (Additional File 1: Table S3). For secondary analysis of ADHD medication, we only included adults who had prior records of an ADHD diagnosis or medication dispensation before or at T2D diagnosis. The study population was followed from the date of T2D diagnosis (start of follow-up) until the outcomes of interest, death, emigration or end of the study period (December 31, 2020, otherwise specified), whichever came first (Additional File 1: Figure S1).

### Measurements

#### Exposure

In the main analysis, the exposure was ADHD, defined as having records of an ADHD diagnosis (ICD-10-code F90) or ADHD medication dispensation (ATC-codes methylphenidate [N06BA04], amphetamine [N06BA01], dexamphetamine [N06BA02], lisdexamfetamine [N06BA12], atomoxetine [N06BA09], guanfacine [C02AC02]). ADHD was regarded as a time-dependent exposure during follow-up (Additional File 1: Figure S2), but once recorded, the ADHD status remained unaltered until the end of study.

In the secondary analysis, ADHD medication was treated as a time-dependent exposure, allowing for multiple on- and off-medication periods during follow-up **(**Additional File 1: Figure S3). On-medication periods were defined as the dispensation date plus the country-specific 75^th^ percentile of the ADHD medication dispensation interval (i.e., time between two consecutive dispensations) among adults of the same sex and age group (young adults[18-34 years], middle-aged adults[35-64 years], elderly[≥65 years]) (Additional File 1: Table S4) [21–23]. Overlapping on-medication periods were ignored (i.e., if two on-medication periods overlapped, the start date of the subsequent dispensation was not moved forward). The intervals between the end of one on-medication period and the start of the next were considered off-medication periods.

#### Outcomes

The outcomes were MACE and all-cause mortality. MACE was a composite measure defined by records of acute myocardial infarction (ICD-10-codes I21-I23), stroke (I60-I64), or CVD-related death (I00-I99 recorded cause of death) [24]. Death records were identified using underlying cause of death.

#### Covariates

Covariates included calendar year and age at baseline (start of follow-up), to adjust for temporal and age-related effects, as well as sex. Psychiatric comorbidity was modeled as a binary variable based on any psychiatric diagnosis within five years before baseline (codes in Additional File 1: Table S2). Psychotropic (excluding ADHD medications) and cardiometabolic medication use were treated as binary variables based on dispensation within three months before baseline **(**codes in Additional File 1: Table S5), to identify current treatment at baseline, thereby reducing misclassification of past or non-active use as ongoing exposure. Antidiabetic medication use (insulin and blood glucose-lowering drugs) was modeled as a time-dependent variable, with on-medication periods defined as the prescription date plus the country-specific 75^th^ percentile of the dispensation interval (codes in Additional File 1: Table S5). Socioeconomic status (SES) was categorized into four levels, where possible, using country-specific income or education measures (Additional File 1: Table S6).

### Statistical analysis

This study applied a distributed network approach using a common data model and standardized analytical protocol to harmonize data and analyses across participating sites [25]. The protocol and analysis plan were preregistered (https://doi.org/10.17605/OSF.IO/FZA9B). We collected baseline characteristics of each study population at the time of T2D diagnosis, stratified by ADHD status at end of follow-up.

To assess associations of ADHD and ADHD medication use with MACE and all-cause mortality, we conducted Cox regression to estimate hazard ratios (HRs) for each exposure separately within countries, using time since T2D diagnosis as the time scale. Models were adjusted sequentially for sex, age and calendar year at T2D diagnosis (Model 1); additionally for psychiatric history, psychotropic and cardiometabolic medication use (Model 2); and additionally antidiabetic medication use during follow-up (Model 3). A false discovery rate adjusted p-value threshold of 0.002, generated using the Benjamini-Hochberg procedure, was used to infer statistical significance [26]. Analyses were stratified by sex and age groups at baseline (18-44; ≥45 years). Country-specific HRs were pooled using random-effects meta-analysis with 95% confidence intervals (CIs). Cross-country heterogeneity was assessed using Cochran’s Q test and the I^2^ statistic, with *p* < 0.05 or I^2^ > 75% indicating significant heterogeneity.

In sensitivity analyses, we conducted several alternative specifications to test the robustness of our findings. First, we redefined the study population using a clinical diagnosis of T2D, excluding individuals identified solely through medication records. Second, we redefined ADHD status based only on a clinical ADHD diagnosis. Third, analyses were restricted to adults younger than 65 years at baseline to assess potential age-related differences. Fourth, we additionally adjusted all models for SES. Fifth, we redefined ADHD status as a time-fixed variable prior to baseline.

Data management and analyses were performed with R (version 4.2.3)

## Results

### Cohort characteristics

After applying exclusion criteria, the baseline data included 1,929,027 adults (909,176 [47.1%] male) with T2D from seven countries between 2010 and 2020 (Additional File 1: Table S7), of whom 44,748 had ADHD (2.3%) at the end of follow-up. Among individuals with ADHD, 33,301 (74.4%) were identified before or at baseline (Additional File 1: Table S8). Among those identified after baseline, the median time from baseline to the first recorded ADHD diagnosis ranged from 2.0 to 5.7 years across countries (Additional File 1: Table S9). The proportion of males was lower in the ADHD than in the non-ADHD group (40.2% vs 47.3%). Adults with ADHD were younger at T2D diagnosis (median range: 34-50 years) than those without ADHD (39-62 years). Across all countries, adults with ADHD had more psychiatric comorbidities and psychotropic medication use than those without ADHD (Table 1). Median follow-up duration ranged from 2.2 to 3.9 years in the ADHD group and from 2.8 to 5.5 years in the non-ADHD group. During follow-up, 120,439 adults (6.2%) experienced incident MACE and 115,945 (6.0%) died. CVD deaths accounted for 7.6%-28.5% of MACE and 2.0%-29.2% of all deaths, except in the US, where CVD deaths represented a markedly higher proportion of all deaths (95.8%) (Additional File 1: Table S10). Baseline characteristics for ADHD medication analyses (Additional File 1: Table S11) and those stratified by ADHD status at cohort entry (Additional File 1: Table S12) are presented in the supplementary materials.

**Table 1:** Characteristics of the study population by country

Characteristics	Australia (n = 342,471)		Denmark (n = 189,690)		Netherlands (n = 237,626)		Norway (n = 19,606)		Sweden (n = 337,605)		United Kingdom (n = 174,032)		United States (n = 627,997)	
	With ADHD	Without ADHD	With ADHD	Without ADHD	With ADHD	Without ADHD	With ADHD	Without ADHD	With ADHD	Without ADHD	With ADHD	Without ADHD	With ADHD	Without ADHD
**No. (%)**^**a**^	3,537 (1.0)	338,934 (99.0)	3,083 (1.6)	186,607 (98.4)	4,288 (1.8)	233,338 (98.2)	677 (3.5)	18,929 (96.5)	5,422 (1.6)	332,183 (98.4)	367 (0.2)	173,665 (99.8)	27,374 (4.4)	600,623 (95.6)
**Male, n (%)**	1,470 (41.6)	145,683 (43.0)	1,405 (45.6)	94,697 (50.7)	2,395 (55.9)	127,545 (54.7)	372 (54.9)	8,835 (46.7)	2,629 (48.5)	173,520 (52.2)	187 (51.0)	89,693 (51.6)	9,553 (34.9)	251,192 (41.8)
**Age at baseline, median [IQR], y**	34 [24,47]	54 [39,66]	41 [30,53]	60 [49,70]	50 [40,59]	62 [52,71]	36 [28,42]	39 [31,44]	41 [30,50]	61 [50,70]	42 [27,57]	59 [48,69]	45 [33,56]	58 [47,66]
**Age groups at baseline, n (%)**														
Early diagnosed T2D (18-44 years)	2,497 (70.6)	112,006 (33.0)	1,778 (57.7)	33,894 (18.2)	1,488 (34.7)	23,463 (10.1)	571 (84.3)	14,402 (76.1)	3,273 (60.4)	50,718 (15.3)	203 (55.3)	32,647 (18.8)	13,263 (48.5)	120,358 (20.0)
Not early diagnosed T2D ( ≥ 45 years)	1,040 (29.4)	226,928 (67.0)	1,305 (42.3)	152,713 (81.8)	2,800 (65.3)	209,875 (89.9)	106 (15.7)	4,527 (23.9)	2,149 (39.6)	281,465 (84.7)	164 (44.7)	141,018 (81.2)	14,111 (51.5)	480,265 (80.0)
**Time to end of follow-up, median [IQR], y**	2.4 [1.1, 4.1]	3.5 [1.7, 5.3]	3.2 [1.2, 5.9]	4.9 [2.1, 8.1]	2.5 [1.1, 4.0]	2.9 [1.4, 4.4]	3.6 [1.4, 6.0]	4.3 [2.0, 7.6]	3.1 [1.3, 5.6]	4.6 [2.1, 7.7]	3.9 [1.6, 6.8]	5.5 [2.6, 8.1]	2.2 [0.9, 4.2]	2.8 [1.2, 5.0]
**Psychiatric comorbidity, n (%)**	1,058 (29.9)	21,301 (6.3)	1,016 (33.0)	12,536 (6.7)	1,839 (42.9)	16,946 (7.3)	222 (32.8)	1,296 (6.8)	3,304 (60.9)	24,855 (7.5)	148 (40.3)	24744 (14.2)	12,133 (44.3)	106,163 (17.7)
**Psychotropic medication use, n (%)**	2,199 (62.2)	80,039 (23.6)	1,487 (48.2)	35,864 (19.2)	582 (13.6)	10,592 (4.5)	393 (58.1)	5,467 (28.9)	3,280 (60.5)	65,883 (19.8)	175 (47.7)	46,380 (26.7)	9,913 (36.2)	88,787 (14.8)
**Cardiometabolic medication use, n (%)**	3,348 (94.7)	317,190 (93.6)	912 (29.6)	96,480 (51.7)	4,288 (100.0)	233,338 (100.0)	43 (6.4)	3,424 (18.1)	1,101 (20.3)	135,379 (40.8)	292 (79.6)	134,046 (77.2)	4,799 (17.5)	104,529 (17.4)

Abbreviations: *T2D* Type 2 diabetes, *ADHD* Attention-deficit/hyperactivity disorder, *MACE* Major adverse cardiac events, *IQR* interquartile range

^a^Adults with and without ADHD by end of follow-up period (for MACE outcome)

### Association of ADHD with MACE and all-cause mortality

**Fig. 1:**
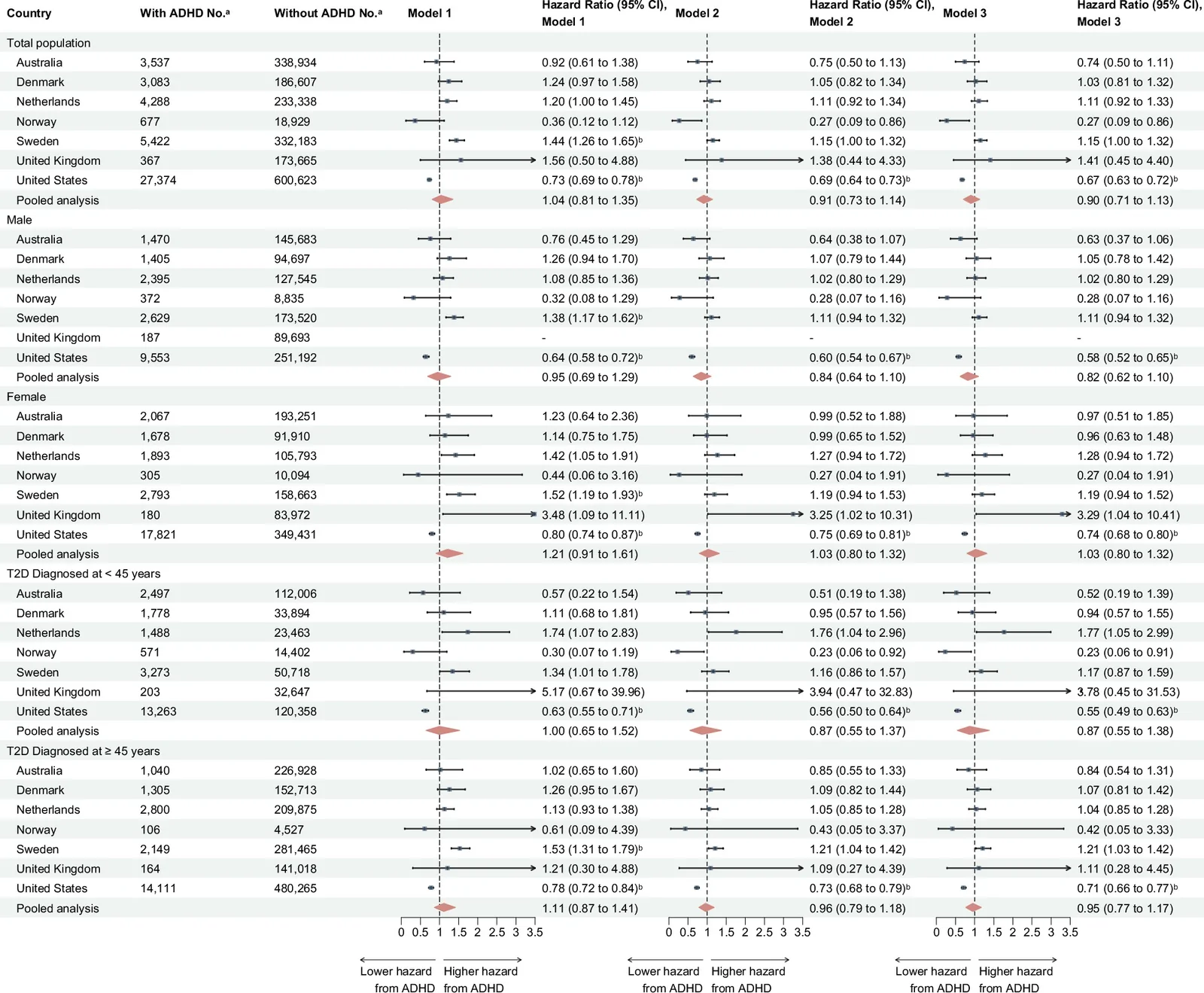
Association between ADHD and incident MACE following T2D diagnosis across countries. Abbreviations: T2D: Type 2 diabetes; ADHD: Attention-deficit/hyperactivity disorder Note: Model 1 adjusted for sex, age and calendar year at T2D diagnosis; Model 2 additionally adjusted for history of psychiatric disorders, psychotropic medication use and cardiometabolic medication use; Model 3 additionally adjusted for antidiabetic medication use during follow-up. Pooled analysis used a random-effects model to pool the effect sizes from all countries. ^a^No. refers to number of adults diagnosed (or not diagnosed) with ADHD or receiving (or not receiving) ADHD medication by study end. ^b^Statistically significant after false discovery rate adjustment

For the pooled data, ADHD did not show an association with MACE (Model 1:HR, 1.04; 95%CI, 0.81-1.35; Fig. 1); the cross-country heterogeneity was significant and high (MACE:*p* < 0.0001, I² = 94.4% [95%CI, 90.9%-96.6%]). In Sweden, ADHD was statistically significantly associated with an increased rate of MACE (HR, 1.44; 95%CI, 1.26-1.65;*p* < 0.002), while in the US, ADHD was associated with a decreased rate of MACE (HR, 0.73; 95%CI, 0.69-0.78;*p* < 0.002). No statistically significant associations were observed in the other countries (HR range, 0.36-1.56; Fig. 1). After further adjusting for psychiatric comorbidities, psychotropic and cardiometabolic medication use in Model 2 and 3, the associations attenuated (Fig. 1).

**Fig. 2:**
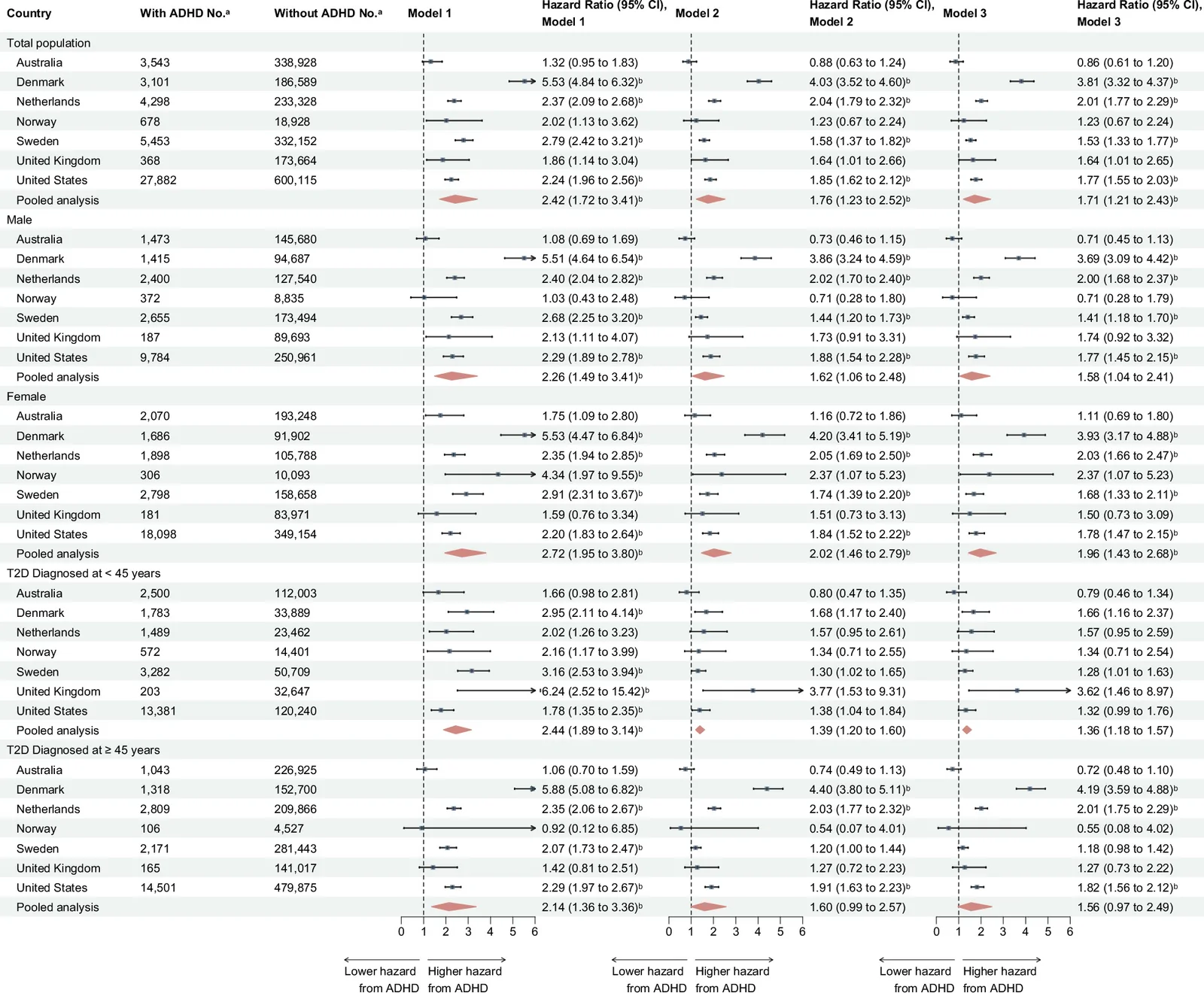
Association between ADHD and all-cause mortality following T2D diagnosis across countries. Abbreviations: T2D: Type 2 diabetes; ADHD: Attention-deficit/hyperactivity disorder Note: Model 1 adjusted for sex, age and calendar year at T2D diagnosis; Model 2 additionally adjusted for history of psychiatric disorders, psychotropic medication use and cardiometabolic medication use; Model 3 additionally adjusted for antidiabetic medication use during follow-up. Pooled analysis used a random-effects model to pool the effect sizes from all countries. ^a^No. refers to number of adults diagnosed (or not diagnosed) with ADHD or receiving (or not receiving) ADHD medication by study end. ^b^Statistically significant after false discovery rate adjustment

The pooled analysis showed more than a twofold increased rate of all-cause mortality (HR, 2.42; 95%CI, 1.72-3.41;*p* < 0.002; Fig. 2) in adults with ADHD compared to those without, after adjusting for sex, age and calendar year at T2D diagnosis (Model 1). The association for all-cause mortality attenuated, but remained significant, when additionally adjusted for psychiatric disorders, psychotropic and cardiometabolic medication use (Model 2:HR, 1.76; 95%CI, 1.23-2.52;*p* < 0.002), and antidiabetic medication use (Model 3:HR, 1.71; 95%CI, 1.21-2.43;*p* < 0.002; Fig. 2). Cross-country heterogeneity was significant and high (*p* < 0.0001,I² = 95.8% [95%CI,93.3-97.3%]). For country-specific analysis, ADHD was statistically significantly associated with increased rate of all-cause mortality across all three models in Sweden, the Netherlands, Denmark and US (Model 3:HR range, 1.53-3.81; Fig. 2).

### Association of ADHD medication with MACE and all-cause mortality

**Fig. 3:**
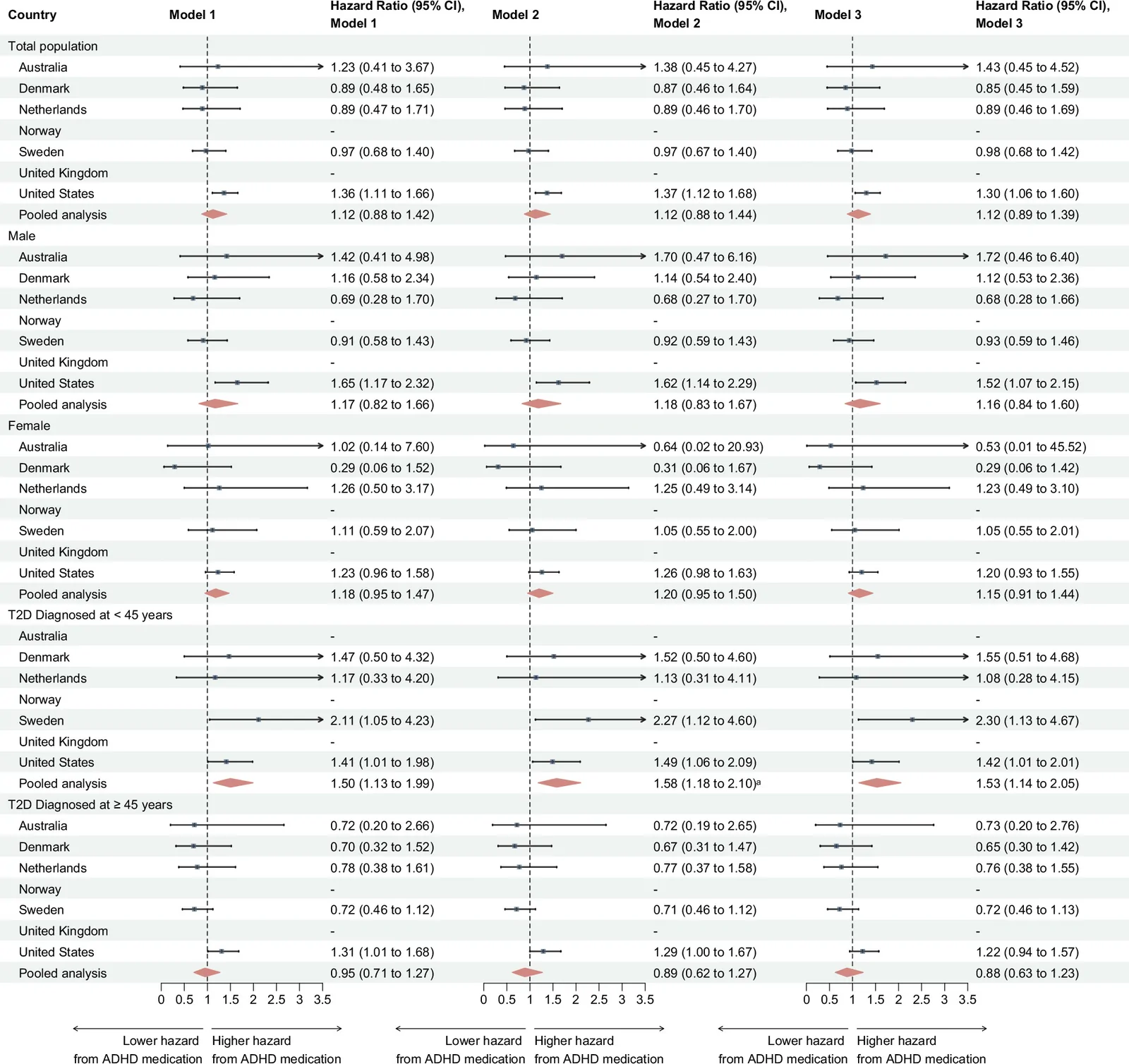
Association between ADHD medication use and incident MACE following T2D diagnosis across countries. Abbreviations: T2D: Type 2 diabetes Note: Model 1 adjusted for sex, age and calendar year at T2D diagnosis; Model 2 additionally adjusted for history of psychiatric disorders, psychotropic medication use and cardiometabolic medication use; Model 3 additionally adjusted for antidiabetic medication use during follow-up. Pooled analysis used a random-effects model to pool the effect sizes from all countries. ^a^Statistically significant after false discovery rate adjustment

**Fig. 4:**
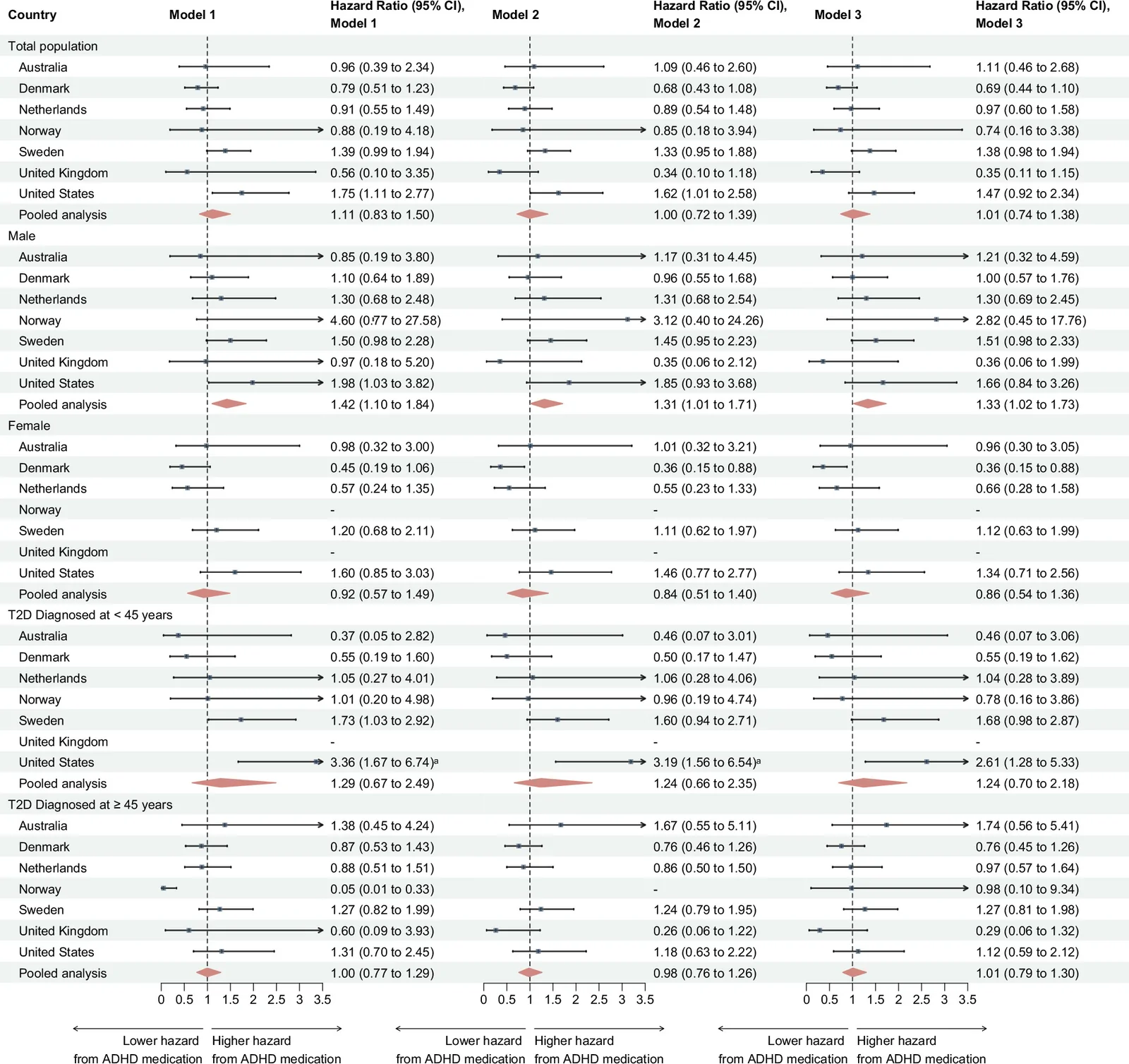
Association between ADHD medication use and all-cause mortality following T2D diagnosis across countries. Abbreviations: T2D: Type 2 diabetes Note: Model 1 adjusted for sex, age and calendar year at T2D diagnosis; Model 2 additionally adjusted for history of psychiatric disorders, psychotropic medication use and cardiometabolic medication use; Model 3 additionally adjusted for antidiabetic medication use during follow-up. Pooled analysis used a random-effects model to pool the effect sizes from all countries. ^a^Statistically significant after false discovery rate adjustment

Among adults with ADHD (n = 33,301), pooled analyses showed no significant associations between ADHD medication use and MACE or all-cause mortality (Fig. 3&4). Cross-country heterogeneity was not significant (MACE:*p* = 0.34,I² = 12% [95%CI,0-81.7%]; all-cause mortality:*p* = 0.18,I² = 33.1% [95%CI,0-71.6%]). Country-specific analyses similarly showed no significant associations for either MACE (HR range, 0.89-1.36) or all-cause mortality (HR range, 0.56-1.75).

### Subgroup analyses

Pooled analyses showed that associations of ADHD with MACE and all-cause mortality were consistent across sex and age groups (Fig. 1&2). Results for ADHD medication were similarly comparable across sex (Fig. 3&4). When stratified by age, a higher MACE rate emerged in the younger group (18-44 years), with a statistically significant association after multiple-testing correction in Model 2 (HR,1.58; 95%CI,1.18-2.10; Fig. 3). Associations with all-cause mortality were consistent across age groups.

### Sensitivity analyses

Due to limited data availability, sensitivity analyses restricted to clinically diagnosed T2D were conducted only in Sweden and the UK. In these analyses, we observed a statistically significant association between ADHD and MACE (Model 1:HR, 1.48;95%CI, 1.24-1.77;*p* < 0.002), although this association was no longer significant after adjustment for comorbidities and medication use (Additional File 1: Table S13). Effect estimates for all-cause mortality were largely unchanged, as reflected by overlapping confidence intervals (Additional File 1: Table S14). In sensitivity analyses where ADHD was defined solely by clinical diagnosis, the effect estimates of MACE and all-cause mortality were comparable to those observed in the main analysis (Additional File 1: Tables S13-S16). Estimates remained consistent after excluding adults with T2D diagnosed after age 65 (Additional File 1: Table S13-16), and after adjustment for SES (Additional File 1: Tables S13-S16). In sensitivity analyses where ADHD was defined prior to baseline, the effect estimates of MACE and all-cause mortality were comparable to those observed in the main analysis (Additional File 1: Tables S13-S14).

## Discussion

In this large multinational cohort study, adults with T2D and co-occurring ADHD had more than twice the rate of all-cause mortality compared to those without ADHD. Although T2D is a well-established risk factor for mortality [9], our findings indicate that ADHD is associated with additional risk. In contrast, we observed no consistent association between ADHD and MACE. ADHD medication use, however, was linked to a 51% higher rate of MACE among adults with early-onset T2D. Together, these findings highlight the complex interplay between ADHD, cardiovascular comorbidity, and mortality in adults with T2D and underscore the need for increased awareness of ADHD in diabetes care and enhanced clinical support to improve health outcomes in this population. While replication is needed, our findings suggest that ADHD medications may need to be prescribed cautiously and monitored closely in adults with T2D and ADHD. There is a need to further investigate why younger individuals may be more vulnerable to CVD related to ADHD medication use, which could help inform targeted preventive strategies.

### Association of ADHD with MACE and all-cause mortality

ADHD was associated with higher all-cause mortality in adults with T2D, consistently across age groups and sex, irrespective of psychiatric comorbidities, psychotropic medication use, and cardiometabolic treatments. Although T2D is a well-established risk factor for mortality [9], our findings suggest that co-occurring ADHD is associated with additional mortality risk. This aligns with prior research showing a 7-9 year reduction in life expectancy in ADHD [27] and more than a twofold increased risk of all-cause mortality, particularly from unnatural causes, such as accidents [12], a magnitude comparable to that observed in our study. ADHD symptoms may increase vulnerability to risky behaviors and unintentional injuries [28], providing a plausible mechanism beyond cardiometabolic pathways. Notably, adjustment for psychiatric comorbidities, psychotropic and cardiometabolic treatments did not fully attenuate the association, suggesting that ADHD could represent an independent mortality risk factor in T2D. Although cause-specific mortality (beyond MACE-related deaths) could not be examined, excess risk may reflect both unnatural causes, as reported in broader ADHD literature, and natural causes related to suboptimal diabetes self-management [19,29]. Cause-specific analyses are warranted to clarify these pathways and inform prevention strategies.

ADHD was not associated with MACE in adults with T2D in pooled analyses, contrasting with previous meta-analytic evidence of elevated CVD risk in the general ADHD population [11]. Point estimates were lower than those reported in general population studies [11], possibly reflecting a ceiling effect whereby the already high cardiovascular risk conferred by T2D may limit the additional risk associated with ADHD [8]. Differences in outcome definitions may further explain inconsistencies, as our study examined MACE specifically, whereas earlier studies examined broader CVD outcomes [11]. As the first study of ADHD and MACE in adults with T2D, replication across different CVD definitions is warranted. Despite pooled null results, country-specific heterogeneity was observed, with significant unadjusted associations in Sweden and the Netherlands. Notably, cross-country differences in ADHD prevalence partly reflects variation in data sources, coverage, and diagnostic practices [30]. In our study, for example, the US data included multiple care settings (e.g., primary and hospital/specialist care), whereas the UK primarily relied on primary care records, potentially leading to under-ascertainment (Additional File 1: Table S1). Such differences may also reflect variation in underlying ADHD populations and contribute to the observed heterogeneity in results.

### Association of ADHD medication with MACE and all-cause mortality

When stratified by age at T2D diagnosis, ADHD medication use was associated with increased MACE risk in younger adults, but not in older adults. This may reflect lower baseline CVD prevalence in younger adults [31], potentially making incident events and potential medication effects more detectable, together with the heightened CVD vulnerability linked to early-onset T2D, driven by accelerated metabolic deterioration and coexisting risk factors such as obesity [32–34]. In older adults, the absence of elevated risk may partly reflect more cautious prescribing practices, including lower starting doses and gradual titration [35]. Unlike studies in the general population reporting similar cardiovascular risks across age groups [14], our findings suggest that age-related differences may be observed in populations with T2D.

To our knowledge, this is the first study investigating ADHD medication in relation to MACE and mortality in individuals with T2D. Although no statistically significant overall cardiovascular harm was observed, stratified analyses suggest a potentially elevated risk in those with early-onset T2D. Prior studies have linked long-term ADHD medication use to increased cardiovascular risk [13,14], and, for atomoxetine, higher T2D risk [36], whereas others reported reduced unnatural-cause mortality following medication initiation [15]. Obesity and unhealthy eating behaviors, which are associated with ADHD [37], and appetite suppression, which is linked with ADHD medication use, may partly contribute to the observed associations, given their established links with CVD and mortality [38]. Future studies should investigate the mediating role of obesity in the relationship between ADHD, its treatment, and cardiovascular outcomes. Additionally, future studies should directly compare CVD and mortality among individuals with ADHD, including populations without T2D, in order to better disentangle the independent and joint contributions of ADHD and metabolic disease to cardiovascular risk and death by natural causes. Larger longitudinal studies with longer follow-up are further needed to clarify age- and medication class-specific cardiovascular risks.

#### Strengths and limitations

By leveraging population-based data across three continents, our findings are likely more generalizable to adults with T2D and ADHD receiving clinical care across healthcare settings. Several limitations warrant consideration. First, detection bias is possible, as individuals with ADHD may have more frequent healthcare interactions, potentially enabling earlier cardiovascular management and delaying progression to MACE. Second, ADHD may be underdiagnosed in older adults, and possible overdiagnosis in younger adults may have biased estimates toward the null. Third, T2D ascertainment likely varied across countries, particularly where primary care data were unavailable, although defining T2D using antidiabetic medication likely captured most cases. Importantly, sensitivity analyses restricted to clinically diagnosed adults with T2D yielded consistent results. MACE definitions are likely more consistent across countries, as these events are typically managed in specialist care, although differences in healthcare systems and diagnostic practices may also have contributed to variation in identified individuals with MACE.

Fourth, ADHD medication analyses were limited by small sample sizes, requiring replication in larger cohorts. Further, exposure definitions based on the 75th percentile refill intervals may not fully capture true medication use, particularly in the presence of overlapping dispensations or irregular refill patterns, and the use of a 3-month pre-baseline window to define psychotropic/cardiometabolic medication use may not fully capture chronic users without recent refills, potentially leading to misclassification. Fifth, findings may partly reflect confounding by indication, as treated individuals are more likely to coincide with more severe ADHD symptoms [39], or conversely, where severe ADHD symptomology leads to difficulties committing to treatment. Furthermore, in some countries (e.g., Denmark and Australia), ADHD ascertainment relied mainly on medication records due to limited specialist/private care data [40,41], precluding comparisons between medicated and non-medicated ADHD groups. Residual confounding or mediation by unmeasured cardiovascular risk factors (e.g., smoking, BMI, blood pressure, glycemic control, renal function, and substance use) cannot be excluded and may partly explain the observed associations. Future studies incorporating more detailed cardiometabolic and lifestyle measures are warranted to better elucidate the relationship between ADHD, medication use, and cardiovascular and mortality outcomes.

Sixth, adjustment for time-varying antidiabetic medication use may have introduced collider bias, as medication use is influenced by both ADHD status and T2D severity. Although antidiabetic medication was included only to assess robustness rather than estimate causal effects, future studies investigating underlying mechanisms should explicitly specify their causal assumptions. Seventh, substantial between-country heterogeneity suggests pooled estimates should be interpreted as average effects across diverse settings rather than single common effects shared across countries. Variation in the direction of estimates, despite overlapping confidence intervals, highlights potential statistical uncertainty as well as differences in healthcare systems, data sources, ADHD ascertainment and underlying age structure across countries, thus, caution is warranted when applying these findings to local settings. Finally, generalizability to low- and middle-income countries may be limited.

## Conclusions

This multinational cohort study indicates that among adults with T2D, ADHD is associated with increased risk of all-cause mortality, but not MACE. The findings further suggest that ADHD medication use in adults with T2D is associated with increased risk of MACE in younger adults. These findings highlight the need for enhanced support for adults with co-occurring T2D and ADHD, and underscore the value of integrated, interdisciplinary care involving healthcare providers from both psychiatric and somatic sectors that may help reduce MACE and mortality risks. Further research is needed to replicate these findings and investigate underlying mechanisms linking ADHD to MACE and mortality in adults with T2D.

## Supplementary information

Supplementary Information

## Data Availability

Australia: Direct access to the data and analytical files to other individuals or authorities is not permitted without the express permission of the approving human research ethics committees and data custodians.Denmark: The data supporting the findings of this study are available from Statistics Denmark. Data access required the completion of a detailed application form from the Danish Data Protection Agency, the Danish National Board of Health, and Statistics Denmark. Researchers at Danish research institutions may obtain the relevant approval and data. International researchers may gain data access if governed by a Danish research institution having needed approval and data access. For more information on accessing the data, see http://www.dst.dk.Netherlands: This study was performed under project agreement 9205 microdata services research. Individual-level data are prohibited from sharing or being publicly available. Data can be obtained by application to Statistics Netherlands.Norway: National regulations and laws prohibit sharing or making individual-level data publicly available. The data can be obtained by application to the respective registries and one of the Norwegian Regional Committeesfor Medical and Health Research Ethics.Sweden: The Public Access to Information and Secrecy Act in Sweden prohibits making individual-level data publicly available. Researchers interested in replicating this work can apply for individual-level data through Statistics Sweden at: https://www.scb.se/en/services/guidance-for-researchers-and-universities/.The United Kingdom: Individual-level data are prohibited from sharing or being publicly available. Data can be obtained by application to the Health Improvement Network.The United States: The individual-level electronic health record data in the TriNetX database cannot be shared, as they are the property of TriNetX and can only be accessed through a direct contract with the company.

## Abbreviations

CI: Confidence interval
ADHD: Attention-deficit/hyperactivity disorder
ATC: Anatomical therapeutic chemical
CVD: Cardiovascular disease
eGFR: Estimated glomerular filtration rate
HbA1c: Haemoglobin A1c
HR: Hazard ratio
ICD: International classification of diseases
MACE: Major adverse cardiac events
SES: Socioeconomic status
STROBE: Strengthening the reporting of observational studies in epidemiology
T1D: Type 1 diabetes
T2D: Type 2 diabetes
UK: United Kingdom
US: United States
