# Supplementary material for: Major adverse cardiac events and mortality associated with ADHD and its treatment in adults with type 2 diabetes: a multi-national cohort study
Source: BMC Med. 2026 Sep 26;24:527. doi: 10.1186/s12916-026-05246-4 (PMC13621677; doi:10.1186/s12916-026-05246-4)
Supplement: Supplementary file 1 [file 12916_2026_5246_MOESM1_ESM.docx]

**Supplemental Online Content**

sMethods. Ethical review and data sharing summary	2

Table S1. Description of data sources for each contributing country	4

Table S2. ICD codes for defining MACE and psychiatric comorbidities	6

Table S3. Exclusion criteria information by country	7

Table S4. The calculated 75th percentile refill intervals for ADHD medications across country, by sex and age group	8

Table S5. ATC codes for defining psychotropic medications and cardiometabolic medications	10

Table S6. Social-economic status variable by country	11

Table S7. Exclusion criteria information by country	12

Table S8. The number and proportion for individuals with ADHD identified before or at baseline versus during follow-up	13

Table S9. The distribution of time from T2D diagnosis to the first ADHD record	14

Table S10. The proportion of CVD death in MACE and all-cause death	15

Table S11. Baseline characteristics among individuals identified as having ADHD before or at baseline by country	16

Table S12. Characteristics by ADHD status at cohort entry by country	17

Table S13. Sensitivity analyses: association between ADHD and incident MACE following T2D diagnosis across countries	18

Table S14. Sensitivity analyses: association between ADHD and all-cause mortality following T2D diagnosis across countries	20

Table S15. Sensitivity analyses: association between ADHD medication use and incident MACE following T2D diagnosis across countries	22

Table S16. Sensitivity analyses: association between ADHD medication use and all-cause mortality following T2D diagnosis across countries	24

Figure S1. Study design	26

Figure S2. ADHD as time-dependent variable	27

Figure S3. Example of ADHD medication use as time-dependent variable	28

References	29

# sMethods. Ethical review and data sharing summary

| **Country** | **Ethical Review Oversight** | **Data sharing statement** |
| --- | --- | --- |
| **Australia** | This research received ethical approval from the AIHW Human Research Ethics Committee (AIHW HREC) (approval number EO2021/1/1233) and the NSW Population and Health Services Research Ethics Committee (PHSREC) (approval number 2020/ETH02273). | Direct access to the data and analytical files to other individuals or authorities is not permitted without the express permission of the approving human research ethics committees and data custodians. |
| **Denmark** | The study was approved by the Danish Data Protection Agency, and data access was agreed upon by Statistics Denmark and the Danish Health Data Authority. Approval by the Ethics Committee and written informed consent were not required for register-based projects (cf. LBK nr 1338 af 01/09/2020, Act no. 1338 of 1 September 2020, section 10 on research ethics for administration of health scientific research projects and health data scientific research projects). | The data supporting the findings of this study are available from Statistics Denmark. Data access required the completion of a detailed application form from the Danish Data Protection Agency, the Danish National Board of Health, and Statistics Denmark. Researchers at Danish research institutions may obtain the relevant approval and data. International researchers may gain data access if governed by a Danish research institution having needed approval and data access. For more information on accessing the data, see http://www.dst.dk. |
| **Netherlands** | Analyses of administrative health records are exempt from the requirement of informed consent. Individual-level data were anonymized and did not leave the Statistics Netherlands server. | This study was performed under project agreement 9205 microdata services research. Individual-level data are prohibited from sharing or being publicly available. Data can be obtained by application to Statistics Netherlands. |
| **Norway** | The study was approved by the Regional Ethics Committee in Western Norway (2020/75421). | National regulations and laws prohibit sharing or making individual-level data publicly available. The data can be obtained by application to the respective registries and one of the Norwegian Regional Committees  for Medical and Health Research Ethics. |
| **Sweden** | This study was approved by the Swedish Ethical Review Authority (2020-06540). Informed consent is not required for pseudonymized register-based research according to Swedish law. | The Public Access to Information and Secrecy Act in Sweden prohibits making individual-level data publicly available. Researchers interested in replicating this work can apply for individual-level data through Statistics Sweden at: https://www.scb.se/en/services/guidance-for-researchers-and-universities/. |
| **The United Kingdom** | The study was approved by the Health Improvement Network Scientific Review Committee (17THIN102). | Individual-level data are prohibited from sharing or being publicly available. Data can be obtained by application to the Health Improvement Network. |
| **The United States** | The study was approved by the Institutional Review Board of the SUNY Upstate Medical University (1933232-2). | The individual-level electronic health record data in the TriNetX database cannot be shared, as they are the property of TriNetX and can only be accessed through a direct contract with the company. |

# Table S1. Description of data sources for each contributing country

|  | **Australia** | **Denmark** | **Netherlands** | **Norway** | **Sweden** | **United Kingdom** | **United States** |
| --- | --- | --- | --- | --- | --- | --- | --- |
| Data source^a^ | MedIntel | registers | registers | registers | registers | THIN | TriNetX |
| Coverage | New South Wales  (Medicare-eligible adults) | nationwide | nationwide | nationwide | nationwide | ~6% of the UK population | nationwide  (76 healthcare organizations) |
| Physical condition diagnosis setting and data availability | hospital (inpatient): 2002-2020 | hospital/specialist (inpatient): 1977-2020  hospital/specialist (outpatient): 1995-2020 | Hospital/specialist (inpatinet/outpatients): 2013-2020 | hospital/specialist (inpatient): 2008-2019 | primary care:1996-2020  hospital/specialist (inpatient): 1964-2020  hospital/specialist (outpatient): 2001-2020 | primary care (GP)^b^:2001-2020 | Primary/hospital/specialist (inpatinet/outpatients): 1979-2020 |
| Psychiatric disorder diagnosis setting and data availability | hospital (inpatient): 2002-2020 | hospital/specialist (inpatient): 1977-2020  hospital/specialist (outpatient): 1995-2020 | Hospital/specialist (inpatinet/outpatients): 2011-2020 | hospital/specialist (inpatient): 2008-2019 | hospital/specialist (inpatient): 1973-2020  hospital/specialist (outpatient): 2001-2020 | primary care (GP)^b^:2001-2020 | Primary/hospital/specialist (inpatinet/outpatients): 1979-2020 |
| Medication setting and data availability | All pharmacies in New South Wales: 2012-07-01 to 2020 | Pharmacies nationwide: 1995-2020 | Pharmacies nationwide: 2006-2020 | Pharmacies nationwide: 2004-2019 | Pharmacies nationwide: 2005-2020 | primary care (GP)^b^: 2001-2020 | Primary/hospital/specialist: 1979-2020 |
| Medication record | dispensation | dispensation | dispensation | dispensation | dispensation | prescription | prescription |
| Diagnosis system (use/mapped to) | ICD | ICD | code mapped to ICD | ICD | ICD | code mapped to ICD | ICD |
| Medication system  (use/mapped to) | ATC | ATC | ATC | ATC | ATC | code mapped to ATC | code mapped to ATC |

Abbreviations: ICD: International statistical classification of diseases; ATC: Anatomical therapeutic chemical; GP: General practice

^a^For Australia, administrative data was linked with register data for Medicare-eligible adults residing in the state of New South Wales (MedIntel Data Platform) [1]; for Denmark, the Netherlands, Norway, and Sweden, data were obtained from registers or electronic health records (EHR) with national coverage [2, 3]; for the UK, primary care data covering 6% of the UK population were obtained from The Health Improvement Network (THIN) medical records database [4] and for the US, data were obtained from federated EHR from the TriNetX Research Network database [5].

^b^Information is routinely recorded on referrals to secondary care, including the specialist care.

# Table S2. ICD codes for defining MACE and psychiatric comorbidities

| **MACE** | **ICD-8 Codes** | **ICD-9 Codes** | **ICD-10 Codes** |
| --- | --- | --- | --- |
| Acute myocardial infarction | 410.0, 410.9 | 410 | I21-I23 |
| Stroke | 430, 431, 433, 434 | 430, 431, 432, 434 | I60, I61, I62, I63, I64 |
| CVD death (death record linked to CVD diagnosis) | 390-458 | 390-459 | I00-I99 |
| **Psychiatric comorbidities** |  |  |  |
| Anxiety Disorder |  |  | F40-F41 |
| Autism spectrum disorders |  |  | F84 |
| Bipolar Disorder |  |  | F30-F31 |
| Depressive disorder |  |  | F32-33 |
| Eating disorders |  |  | F50 |
| Intellectual disability |  |  | F70-F79 |
| Personality disorder |  |  | F60-F61 |
| Schizophrenia spectrum disorder |  |  | F20-F29 |
| Substance use disorder |  |  | F10-16, F18-F19 |

Abbreviations: MACE: Major adverse cardiac events; CVD: cardiovascular disease

# Table S3. Exclusion criteria information by country

| **Selection criteria** | **Australia** | **Denmark** | **Netherlands** | **Norway** | **Sweden** | **United Kingdom** | **United States** |
| --- | --- | --- | --- | --- | --- | --- | --- |
| Death information | ✓ | ✓ | ✓ | ✓ | ✓ | ✓ | ✓ |
| Emigration information | X | ✓ | ✓ | ✓ | ✓ | ✓ | X^*^ |
| Clinical diagnosis of T1D | X | ✓ | X | ✓ | ✓ | ✓ | ✓ |
| Prescription of insulin | ✓ | ✓ | ✓ | ✓ | ✓ | ✓ | ✓ |

Abbreviations: T1D: Type 1 diabetes

Note: √ = Required data available; X = Required data unavailable; X* = Use last visit record as proxy of emigration

# Table S4. The calculated 75th percentile refill intervals for ADHD medications across country, by sex and age group

| **Country** | **Sex** | **Age category** | **75th percentile refill intervals for ADHD medications (days)** |
| --- | --- | --- | --- |
| Australia | Male | Young adults | 38 |
| Netherlands | Male | Young adults | 29 |
| Sweden | Male | Young adults | 28 |
| United Kingdom | Male | Young adults | 42 |
| United States | Male | Young adults | 35 |
| Denmark | Male | Young adults | 44 |
| Norway | Male | Young adults | 61 |
| Australia | Female | Young adults | 46 |
| Netherlands | Female | Young adults | 33 |
| Sweden | Female | Young adults | 33 |
| United Kingdom | Female | Young adults | 30 |
| United States | Female | Young adults | 35 |
| Denmark | Female | Young adults | 47 |
| Norway | Female | Young adults | 62 |
| Australia | Male | Middle-aged adults | 43 |
| Netherlands | Male | Middle-aged adults | 39 |
| Sweden | Male | Middle-aged adults | 30 |
| United Kingdom | Male | Middle-aged adults | 29 |
| United States | Male | Middle-aged adults | 36 |
| Denmark | Male | Middle-aged adults | 44 |
| Norway | Male | Middle-aged adults | 61 |
| Australia | Female | Middle-aged adults | 45 |
| Netherlands | Female | Middle-aged adults | 35 |
| Sweden | Female | Middle-aged adults | 33 |
| United Kingdom | Female | Middle-aged adults | 54 |
| United States | Female | Middle-aged adults | 37 |
| Denmark | Female | Middle-aged adults | 42 |
| Norway | Female | Middle-aged adults | 61 |
| Australia | Male | The elderly | 38 |
| Netherlands | Male | The elderly | 21 |
| Sweden | Male | The elderly | 33 |
| United Kingdom | Male | The elderly | 40 |
| United States | Male | The elderly | 37 |
| Denmark | Male | The elderly | 64 |
| Norway | Male | The elderly | NA |
| Australia | Female | The elderly | 48 |
| Netherlands | Female | The elderly | 14 |
| Sweden | Female | The elderly | 85 |
| United Kingdom | Female | The elderly | NA |
| United States | Female | The elderly | 35 |
| Denmark | Female | The elderly | 70 |
| Norway | Female | The elderly | NA |

Note: young adults[18-34 years], middle-aged adults[35-64 years], the elderly[≥65 years]

# Table S5. ATC codes for defining psychotropic medications and cardiometabolic medications

| **Psychotropic medications** |  | **ATC Codes^a^** |
| --- | --- | --- |
| Antiepileptics |  | N03A |
| Antipsychotics |  | N05A |
| Anxiolytics |  | N05B |
| Hypnotics and sedatives |  | N05C |
| Antidepressants |  | N06A |
| Anti-addiction agents |  | N07B |
| **Cardiometabolic medications** | **Cardiometabolic disease category** |  |
| insulin | Diabetes | A10A |
| blood glucose lowering drugs (excl. insulin) |  | A10B |
| lipid modifying agent | Hyperlipidemia | C10 |
| thiazide | Hypertension | C03A |
| sulfonamide low-ceiling diuretic |  | C03BA |
| other potassium sparing agent |  | C03DB |
| low-ceiling + potassium-sparing |  | C03EA |
| ACEi + diuretic |  | C09BA |
| ARB + diuretic |  | C09DA |
| methyldopa |  | C02AB |
| hydrazinophthalazine derivative |  | C02DB |
| high-ceiling diuretic |  | C03CA, C03CB, C03CC |
| CCB, vascular |  | C08C |
| CCB + diuretic |  | C08G |
| ACEi or ARB |  | C09A, C09C |
| ACEi + CCB |  | C09BB |
| ARB + CCB |  | C09DB |
| ARBs, other combinations |  | C09DX |
| vasodilators | Ischemic heart disease | C01D |
| other non-selective CCB |  | C08EX |
| beta-blocking agent, selective |  | C07AB |
| alpha- and beta-blocking agent |  | C07AG |
| CCB, cardiac |  | C08D |

Abbreviations: ACEi: angiotensin-converting enzyme inhibitor; ARB: angiotensin receptor blocker; CCB: calcium channel blocker

^a^ATC lists for cardiometabolic medications are modified from RxRisk-V Category [6]

# Table S6. Social-economic status variable by country

| **Country** | **Social-economic status variable** | **Data availability** |
| --- | --- | --- |
| Australia | Index of relative social disadvantage (Socio-Economic Indexes for Areas [SEIFA]) | 2002-2020 |
| Denmark | Highest education attainment | 1981-2020 |
| Netherlands | Personal gross income | 2006-2020 |
| Norway | Highest education attainment | 1985-2019 |
| Sweden | Highest education attainment | 1990-2020 |
| United Kingdom | Area material deprivation (Townsend Index) | 2001-2020 |
| United States | Housing or education/employment issues (ICD-10 codes Z55-Z65) | 1979-2020 |

# Table S7. Exclusion criteria information by country

| **Selection criteria** | **Australia** | **Denmark** | **Netherlands** | **Norway** | **Sweden** | **United Kingdom** | **United States** | **Total** |
| --- | --- | --- | --- | --- | --- | --- | --- | --- |
| All individuals identified as T2D between 2010 to 2020 | 376414 | 251581 | 270920 | 26652 | 482856 | 186009 | 737108 | 2331540 |
| After excluding individuals younger than 18 years at baseline | 370931 | 250819 | 270160 | 25898 | 480335 | 185127 | 736470 | 2319740 |
| After excluding individuals emigrating before or at baseline | 370931 | 228328 | 253712 | 24800 | 458677 | 185127 | 722038 | 2243613 |
| After excluding individuals died before or at baseline | 370525 | 228242 | 253700 | 24782 | 458544 | 185127 | 719959 | 2240879 |
| After excluding individuals identified as T1D before or at baseline | 363074 | 215626 | 253700 | 20133 | 432727 | 179773 | 667333 | 2132366 |
| After excluding individuals who had MACE before or at baseline | 342471 | 189690 | 237626 | 19606 | 337605 | 174032 | 627997 | 1929027 |
| Individuals who met the eligibility criteria | 342471 | 189690 | 237626 | 19606 | 337605 | 174032 | 627997 | 1929027 |

# Table S8. Number and proportion of individuals with ADHD identified before/at baseline versus during follow-up

| **Country** | **All ADHD, No.** | **ADHD before/at baseline, No. (%)** | **ADHD during follow-up, No. (%)** |
| --- | --- | --- | --- |
| Australia | 3,537 | 2,389 (67.5%) | 1,148 (32.5%) |
| Denmark | 3,083 | 2,189 (71.0%) | 894 (29.0%) |
| Netherlands | 4,288 | 3,558 (83.0%) | 730 (17.0%) |
| Norway | 677 | 495 (73.1%) | 182 (26.9%) |
| Sweden | 5,422 | 4,000 (73.8%) | 1,422 (26.2%) |
| United Kingdom | 367 | 283 (77.1%) | 84 (22.9%) |
| United States | 27,374 | 20,387 (74.5%) | 6,987 (25.5%) |
| **Total** | **44,748** | **33,301** (**74.4%**) | **11,447** (**25.6%**) |

# Table S9. Distribution of time from T2D diagnosis to the first ADHD record

| **Country** | **Time from baseline to first recorded ADHD, median [IQR], y** |
| --- | --- |
| Australia | 3.1 [1.6, 5.0] |
| Denmark | 3.7 [1.6, 7.0] |
| Netherlands | 2.0 [1.0, 3.6] |
| Norway | 5.7 [2.8, 8.2] |
| Sweden | 3.9 [1.8, 7.0] |
| United Kingdom | 3.9 [1.8, 6.2] |
| United States | 2.3 [1.0, 4.4] |

Note: For the individuals with first ADHD records after T2D diagnosis

# Table S10. Proportion of CVD deaths among MACE and all-cause deaths

| **Country** | **CVD deaths among MACE, %** | **CVD deaths among all-cause deaths, %** |
| --- | --- | --- |
| Australia | 24.5 | 20.9 |
| Denmark | 28.5 | 20.4 |
| Netherlands | 25.4 | 20.4 |
| Norway | 19.5 | 29.2 |
| Sweden | 16.2 | 26.2 |
| United Kingdom | 10.6 | 2.0 |
| United States | 7.6 | 95.8 |
| **Total** | **16.1** | **23.4** |

Abbreviations: CVD: cardiovascular diseases; MACE: Major adverse cardiac events

# Table S11. Baseline characteristics among individuals identified as having ADHD before or at baseline by country

| **Baseline characteristics** | **Australia** | **Denmark** | **Netherlands** | **Norway** | **Sweden** | **United Kingdom** | **United States** |
| --- | --- | --- | --- | --- | --- | --- | --- |
| N | 2,389 | 2,189 | 3,558 | 495 | 4,000 | 283 | 20,387 |
| Age, median [IQR], y | 34 [24, 47] | 41 [29,51] | 49 [40, 58] | 37 [28, 43] | 41 [30, 51] | 38 [26, 53] | 44 [32, 56] |
| Age groups, n (%) |  |  |  |  |  |  |  |
| Early diagnosed T2D (18-44 years) | 1,652 (69.2) | 1,291 (59.0) | 1,248 (35.1) | 394 (79.6) | 2,345 (58.6) | 176 (62.2) | 10,223 (50.1) |
| Not early diagnosed T2D (≥45 years) | 737 (30.8) | 898 (41.0) | 2310 (64.9) | 101 (20.4) | 1655 (41.4) | 107 (37.8) | 10164 (49.9) |
| Male, n (%) | 1075 (45.0) | 1030 (47.1)^a^ | 1968 (55.3) | 302 (61.0) | 2009 (50.2) | 146 (51.6) | 7200 (35.3) |
| Psychiatric comorbidity, n (%) | 760 (31.8) | 829 (37.9) | 1602 (45.0) | 209 (42.2) | 2689 (67.2) | 127 (44.9) | 10087 (49.5) |
| Psychotropic medication use, n (%) | 1490 (62.4) | 1105 (50.5) | 507 (14.2) | 311 (62.8) | 2510 (62.7) | 138 (48.8) | 8005 (39.3) |
| Cardiometabolic medication use, n (%) | 2254 (94.3) | 647 (29.6) | 3558 (100.0) | 24 (4.8) | 861 (21.5) | 216 (76.3) | 3877 (19.0) |

Abbreviations: T2D: Type 2 diabetes; ADHD: Attention-deficit/hyperactivity disorder; IQR: interquartile range

^a^Originally a range given as 1025- 1034, due to local data regulation

# Table S12. Characteristics by ADHD status at cohort entry by country

| **Characteristics** | **Australia**  **(n=342,471)** | | **Denmark**  **(n=189,690)** | | **Netherlands**  **(n=237,626)** | | **Norway**  **(n=19,606)** | | **Sweden**  **(n=337,605)** | | **United Kingdom**  **(n=174,032)** | | **United States**  **(n=627,997)** | |
| --- | --- | --- | --- | --- | --- | --- | --- | --- | --- | --- | --- | --- | --- | --- |
|  | **With ADHD** | **Without ADHD** | **With ADHD** | **Without ADHD** | **With ADHD** | **Without ADHD** | **With ADHD** | **Without ADHD** | **With ADHD** | **Without ADHD** | **With ADHD** | **Without ADHD** | **With ADHD** | **Without ADHD** |
| **No. (%)^a^** | 2,389 (0.7) | 340,082 (99.3) | 2,189 (1.2) | 187,501 (98.8) | 3,558 (1.5) | 234,068 (98.5) | 495 (2.5) | 19,111 (97.5) | 4,000 (1.2) | 333,605 (98.8) | 283 (0.2) | 173,749 (99.8) | 20,387 (3.2) | 607,610 (96.8) |
| **Male, n (%)** | 1075 (45.0) | 146079 (43.0) | 1030 (47.1) | 95072 (50.7) | 1968 (55.3) | 127972 (54.7) | 302 (61.0) | 8905 (46.6) | 2009 (50.2) | 174140 (52.2) | 146 (51.6) | 89734 (51.6) | 7200 (35.3) | 253545 (41.7) |
| **Age at baseline, median [IQR], y** | 34 [24, 47] | 54 [39, 66] | 41 [29,51] | 60 [49,70] | 49 [40, 58] | 62 [52, 71] | 37 [28, 43] | 39 [31, 44] | 41 [30, 51] | 61 [50, 70] | 38 [26, 53] | 59 [48, 69] | 44 [32, 56] | 57 [47, 66] |
| **Age groups at baseline, n (%)** |  |  |  |  |  |  |  |  |  |  |  |  |  |  |
| Early diagnosed T2D (18-44 years) | 1,652 (69.2) | 112,851 (33.2) | 1,291 (59.0) | 34,381 (18.3) | 1,248 (35.1) | 23,703 (10.1) | 394 (79.6) | 14,579 (76.3) | 2,345 (58.6) | 51,646 (15.5) | 176 (62.2) | 32,674 (18.8) | 10,223 (50.1) | 123,398 (20.3) |
| Not early diagnosed T2D (≥45 years) | 737 (30.8) | 227231 (66.8) | 898 (41.0) | 153120 (81.7) | 2310 (64.9) | 210365 (89.9) | 101 (20.4) | 4532 (23.7) | 1655 (41.4) | 281959 (84.5) | 107 (37.8) | 141075 (81.2) | 10164 (49.9) | 484212 (79.7) |
| **Psychiatric comorbidity, n (%)** | 760 (31.8) | 21599 (6.4) | 829 (37.9) | 12723 (6.8) | 1602 (45.0) | 17183 (7.3) | 209 (42.2) | 1309 (6.8) | 2689 (67.2) | 25470 (7.6) | 127 (44.9) | 24765 (14.3) | 10087 (49.5) | 108209 (17.8) |
| **Psychotropic medication use, n (%)** | 1490 (62.4) | 80748 (23.7) | 1105 (50.5) | 36246 (19.3) | 507 (14.2) | 10667 (4.6) | 311 (62.8) | 5549 (29.0) | 2510 (62.7) | 66653 (20.0) | 138 (48.8) | 46417 (26.7) | 8005 (39.3) | 90695 (14.9) |
| **Cardiometabolic medication use, n (%)** | 2254 (94.3) | 318284 (93.6) | 647 (29.6) | 96745 (51.6) | 3558 (100.0) | 234068 (100.0) | 24 (4.8) | 3443 (18.0) | 861 (21.5) | 135619 (40.7) | 216 (76.3) | 134122 (77.2) | 3877 (19.0) | 105451 (17.4) |

Abbreviations: T2D: Type 2 diabetes; ADHD: Attention-deficit/hyperactivity disorder; MACE: Major adverse cardiac events; IQR: interquartile range

^a^Adults with and without ADHD by end of follow-up period (for MACE outcome)

# Table S13. Sensitivity analyses: association between ADHD and incident MACE following T2D diagnosis across countries

| **Model** | **Australia** | **Denmark** | **Netherlands** | **Norway** | **Sweden** | **United Kingdom** | **United States** | **Pooled analysis** |
| --- | --- | --- | --- | --- | --- | --- | --- | --- |
| **Redefine T2D by clinical diagnosis** |  |  |  |  |  |  |  |  |
| Model 1 | - | - | - | - | 1.48 (1.24 to 1.77)^a^ | 1.57 (0.50 to 4.93) | - | 1.48 (1.24 to 1.77)^a^ |
| Model 2 | - | - | - | - | 1.15 (0.96 to 1.38) | 1.40 (0.45 to 4.40) | - | 1.16 (0.97 to 1.38) |
| Model 3 | - | - | - | - | 1.15 (0.96 to 1.38) | 1.40 (0.44 to 4.40) | - | 1.16 (0.97 to 1.38) |
| **Redefine ADHD by clinical diagnosis** |  |  |  |  |  |  |  |  |
| Model 1 | - | 1.23 (0.74 to 2.05) | 1.61 (1.19 to 2.19) | 0.15 (0.02 to 1.08) | 1.55 (1.33 to 1.80) ^a^ | 0.63 (0.09 to 4.49) | 0.12 (0.10 to 0.15)^a^ | 0.63 (0.23 to 1.71) |
| Model 2 | - | 0.93 (0.56 to 1.55) | 1.42 (1.04 to 1.93) | 0.11 (0.02 to 0.79) | 1.21 (1.04 to 1.41) | 0.57 (0.08 to 4.03) | 0.11 (0.09 to 0.14)^a^ | 0.52 (0.20 to 1.37) |
| Model 3 | - | 0.91 (0.55 to 1.52) | 1.41 (1.04 to 1.93) | 0.11 (0.02 to 0.79) | 1.21 (1.04 to 1.41) | 0.58 (0.08 to 4.08) | 0.11 (0.09 to 0.14)^a^ | 0.52 (0.20 to 1.36) |
| **Restrict study population to ≤65 years** |  |  |  |  |  |  |  |  |
| Model 1 | 0.70 (0.43 to 1.14) | 1.03 (0.78 to 1.36) | 1.15 (0.91 to 1.44) | 0.36 (0.12 to 1.12) | 1.32 (1.14 to 1.53)^a^ | 1.77 (0.44 to 7.17) | 0.69 (0.64 to 0.74)^a^ | 0.94 (0.72 to 1.22) |
| Model 2 | 0.59 (0.36 to 0.96) | 0.86 (0.65 to 1.14) | 1.06 (0.84 to 1.34) | 0.27 (0.09 to 0.86) | 1.07 (0.92 to 1.24) | 1.44 (0.36 to 5.85) | 0.64 (0.59 to 0.69)^a^ | 0.81 (0.64 to 1.04) |
| Model 3 | 0.59 (0.36 to 0.96) | 0.85 (0.64 to 1.13) | 1.06 (0.84 to 1.34) | 0.27 (0.09 to 0.86) | 1.07 (0.92 to 1.24) | 1.49 (0.37 to 6.02) | 0.62 (0.58 to 0.67)^a^ | 0.80 (0.62 to 1.04) |
| **Additionally adjusted for SES** |  |  |  |  |  |  |  |  |
| Model 1 | 0.97 (0.64 to 1.46) | 1.20 (0.93 to 1.54) | 1.19 (0.99 to 1.43) | 0.32 (0.10 to 1.00) | 1.47 (1.29 to 1.69)^a^ | 2.27 (0.31 to 16.64) | 0.69 (0.64 to 0.74)^a^ | 1.02 (0.77 to 1.36) |
| Model 2 | 0.79 (0.53 to 1.19) | 1.02 (0.79 to 1.31) | 1.10 (0.91 to 1.33) | 0.26 (0.08 to 0.81) | 1.17 (1.01 to 1.34) | 2.21 (0.30 to 16.18) | 0.66 (0.62 to 0.71)^a^ | 0.90 (0.70 to 1.14) |
| Model 3 | 0.78 (0.52 to 1.18) | 1.00 (0.78 to 1.28) | 1.10 (0.91 to 1.33) | 0.26 (0.08 to 0.81) | 1.16 (1.01 to 1.34) | 2.40 (0.33 to 17.58) | 0.64 (0.60 to 0.69)^a^ | 0.89 (0.69 to 1.14) |
| **Redefine ADHD using baseline status** |  |  |  |  |  |  |  |  |
| Model 1 | 0.74 (0.44 to 1.24) | 1.26 (0.96 to 1.66) | 1.15 (0.94 to 1.40) | 0.49 (0.16 to 1.52) | 1.52 (1.31 to 1.77)^a^ | 0.67 (0.09 to 4.77) | 0.62 (0.57 to 0.67)^a^ | 0.96 (0.69 to 1.33) |
| Model 2 | 0.75 (0.50 to 1.13) | 1.05 (0.82 to 1.34) | 1.11 (0.92 to 1.34) | 0.27 (0.09 to 0.86) | 1.15 (1.00 to 1.32) | 1.38 (0.44 to 4.33) | 0.69 (0.64 to 0.73)^a^ | 0.91 (0.73 to 1.14) |
| Model 3 | 0.74 (0.50 to 1.11) | 1.03 (0.81 to 1.32) | 1.11 (0.92 to 1.33) | 0.27 (0.09 to 0.86) | 1.15 (1.00 to 1.32) | 1.41 (0.45 to 4.40) | 0.67 (0.63 to 0.72)^a^ | 0.90 (0.71 to 1.13) |

Abbreviations: T2D: Type 2 diabetes; ADHD: Attention-deficit/hyperactivity disorder; SES: Socioeconomic status

Note: Model 1 adjusted for sex, age and calendar year at T2D identification; Model 2 additionally adjusted for history of psychiatric disorders, psychotropic medication use and cardiometabolic medication use; Model 3 additionally adjusted for antidiabetic medication use during follow-up. Pooled analysis used a random-effects model to pool the effect sizes from all countries with available results.

^a^Statistically significant after false discovery rate adjustment

# Table S14. Sensitivity analyses: association between ADHD and all-cause mortality following T2D diagnosis across countries

| **Model** | **Australia** | **Denmark** | **Netherlands** | **Norway** | **Sweden** | **United Kingdom** | **United States** | **Pooled analysis** |
| --- | --- | --- | --- | --- | --- | --- | --- | --- |
| **Redefine T2D by clinical diagnosis** |  |  |  |  |  |  |  |  |
| Model 1 | - | - | - | - | 2.70 (2.28 to 3.20)^a^ | 1.28 (0.63 to 2.62) | - | 2.02 (0.99 to 4.12) |
| Model 2 | - | - | - | - | 1.52 (1.28 to 1.81)^a^ | 1.17 (0.59 to 2.32) | - | 1.50 (1.26 to 1.77)^a^ |
| Model 3 | - | - | - | - | 1.51 (1.27 to 1.79)^a^ | 1.15 (0.57 to 2.32) | - | 1.49 (1.26 to 1.76)^a^ |
| **Redefine ADHD by clinical diagnosis** |  |  |  |  |  |  |  |  |
| Model 1 | - | 2.34 (1.62 to 3.38)^a^ | 1.02 (0.70 to 1.49) | 1.91 (0.98 to 3.72) | 3.00 (2.55 to 3.52)^a^ | 1.58 (0.89 to 2.78) | 1.76 (1.43 to 2.18)^a^ | 1.84 (1.33 to 2.54)^a^ |
| Model 2 | - | 1.28 (0.89 to 1.84) | 0.78 (0.53 to 1.14) | 1.13 (0.57 to 2.24) | 1.57 (1.33 to 1.85)^a^ | 1.42 (0.81 to 2.49) | 1.42 (1.15 to 1.76)^a^ | 1.28 (1.02 to 1.59) |
| Model 3 | - | 1.23 (0.86 to 1.77) | 0.77 (0.52 to 1.13) | 1.13 (0.57 to 2.23) | 1.53 (1.29 to 1.80)^a^ | 1.42 (0.82 to 2.48) | 1.38 (1.12 to 1.71) | 1.26 (1.01 to 1.56) |
| **Restrict study population to ≤65 years** |  |  |  |  |  |  |  |  |
| Model 1 | 1.22 (0.85 to 1.75) | 4.21 (3.62 to 4.90)^a^ | 2.03 (1.69 to 2.43)^a^ | 2.02 (1.13 to 3.62) | 2.25 (1.94 to 2.60)^a^ | 1.91 (0.94 to 3.89) | 1.96 (1.68 to 2.30)^a^ | 2.18 (1.57 to 3.03)^a^ |
| Model 2 | 0.70 (0.49 to 1.01) | 2.71 (2.32 to 3.17)^a^ | 1.70 (1.40 to 2.05)^a^ | 1.23 (0.67 to 2.24) | 1.13 (0.97 to 1.31) | 1.47 (0.72 to 3.00) | 1.60 (1.36 to 1.88)^a^ | 1.46 (1.01 to 2.12) |
| Model 3 | 0.69 (0.48 to 1.00) | 2.63 (2.25 to 3.08)^a^ | 1.68 (1.39 to 2.03)^a^ | 1.23 (0.67 to 2.24) | 1.12 (0.96 to 1.30) | 1.49 (0.73 to 3.04) | 1.52 (1.30 to 1.79)^a^ | 1.44 (1.00 to 2.08) |
| **Additionally adjusted for SES** |  |  |  |  |  |  |  |  |
| Model 1 | 1.35 (0.97 to 1.88) | 5.66 (4.97 to 6.45)^a^ | 2.37 (2.09 to 2.68)^a^ | 1.62 (0.91 to 2.89) | 2.81 (2.44 to 3.23)^a^ | 1.88 (0.67 to 5.33) | 2.20 (1.92 to 2.52)^a^ | 2.36 (1.69 to 3.30)^a^ |
| Model 2 | 0.91 (0.65 to 1.27) | 4.08 (3.57 to 4.67)^a^ | 2.04 (1.79 to 2.32)^a^ | 1.11 (0.61 to 2.03) | 1.58 (1.36 to 1.82)^a^ | 1.81 (0.65 to 5.07) | 1.86 (1.62 to 2.13)^a^ | 1.74 (1.21 to 2.51) |
| Model 3 | 0.88 (0.63 to 1.24) | 3.85 (3.35 to 4.43)^a^ | 2.01 (1.77 to 2.29)^a^ | 1.11 (0.61 to 2.03) | 1.53 (1.32 to 1.76)^a^ | 1.88 (0.68 to 5.19) | 1.77 (1.55 to 2.04)^a^ | 1.70 (1.19 to 2.41) |
| **Redefine ADHD using baseline status** |  |  |  |  |  |  |  |  |
| Model 1 | 1.21 (0.82 to 1.78) | 2.02 (1.65 to 2.48)^a^ | 1.39 (1.19 to 1.64)^a^ | 2.21 (1.17 to 4.18) | 2.62 (2.22 to 3.09)^a^ | 1.79 (0.99 to 3.22) | 1.63 (1.37 to 1.95)^a^ | 1.78 (1.43 to 2.21)^a^ |
| Model 2 | 0.88 (0.63 to 1.24) | 4.03 (3.52 to 4.60)^a^ | 2.04 (1.79 to 2.32)^a^ | 1.23 (0.67 to 2.24) | 1.58 (1.37 to 1.82)^a^ | 1.64 (1.01 to 2.66) | 1.85 (1.62 to 2.12)^a^ | 1.76 (1.23 to 2.52) |
| Model 3 | 0.86 (0.61 to 1.20) | 3.81 (3.32 to 4.37)^a^ | 2.01 (1.77 to 2.29)^a^ | 1.23 (0.67 to 2.24) | 1.53 (1.33 to 1.77)^a^ | 1.64 (1.01 to 2.65) | 1.77 (1.55 to 2.03)^a^ | 1.71 (1.21 to 2.43) |

Abbreviations: T2D: Type 2 diabetes; ADHD: Attention-deficit/hyperactivity disorder; SES: Socioeconomic status

Note: Model 1 adjusted for sex, age and calendar year at T2D identification; Model 2 additionally adjusted for history of psychiatric disorders, psychotropic medication use and cardiometabolic medication use; Model 3 additionally adjusted for antidiabetic medication use during follow-up. Pooled analysis used a random-effects model to pool the effect sizes from all countries with available results.

^a^Statistically significant after false discovery rate adjustment

# Table S15. Sensitivity analyses: association between ADHD medication use and incident MACE following T2D diagnosis across countries

| **Analysis** | **Australia** | **Denmark** | **Netherlands** | **Norway** | **Sweden** | **United Kingdom** | **United States** | **Pooled analysis** |
| --- | --- | --- | --- | --- | --- | --- | --- | --- |
| **Redefine T2D by clinical diagnosis** |  |  |  |  |  |  |  |  |
| Model 1 | - | - | - | - | 0.96 (0.61 to 1.51) | - | - | - |
| Model 2 | - | - | - | - | 0.96 (0.61 to 1.52) | - | - | - |
| Model 3 | - | - | - | - | 0.99 (0.63 to 1.56) | - | - | - |
| **Redefine ADHD by clinical diagnosis** |  |  |  |  |  |  |  |  |
| Model 1 | - | 0.45 (0.12 to 1.76) | 1.04 (0.47 to 2.32) | - | 1.03 (0.69 to 1.53) | - | 1.80 (0.96 to 3.37) | 1.13 (0.80 to 1.60) |
| Model 2 | - | 0.43 (0.10 to 1.85) | 1.09 (0.48 to 2.46) | - | 1.03 (0.69 to 1.54) | - | 1.53 (0.80 to 2.90) | 1.09 (0.80 to 1.49) |
| Model 3 | - | 0.43 (0.10 to 1.82) | 1.09 (0.48 to 2.49) | - | 1.05 (0.70 to 1.57) | - | 1.40 (0.74 to 2.65) | 1.08 (0.80 to 1.48) |
| **Restrict study population to ≤65 years** |  |  |  |  |  |  |  |  |
| Model 1 | 2.44 (0.72 to 8.26) | 0.74 (0.36 to 1.54) | 1.05 (0.52 to 2.10) | - | 1.01 (0.70 to 1.48) | - | 1.27 (1.02 to 1.58) | 1.17 (0.97 to 1.40) |
| Model 2 | 2.66 (0.85 to 8.31) | 0.73 (0.34 to 1.56) | 1.04 (0.51 to 2.10) | - | 1.02 (0.70 to 1.50) | - | 1.30 (1.04 to 1.61) | 1.19 (0.98 to 1.45) |
| Model 3 | 2.73 (0.83 to 8.96) | 0.71 (0.34 to 1.51) | 1.06 (0.51 to 2.18) | - | 1.03 (0.70 to 1.51) | - | 1.24 (0.99 to 1.54) | 1.16 (0.97 to 1.39) |
| **Additionally adjusted for SES** |  |  |  |  |  |  |  |  |
| Model 1 | 1.23 (0.41 to 3.64) | 0.86 (0.45 to 1.64) | 0.90 (0.48 to 1.71) | - | 0.98 (0.68 to 1.41) | - | 1.41 (1.15 to 1.72)^a^ | 1.13 (0.87 to 1.46) |
| Model 2 | 1.39 (0.45 to 4.36) | 0.87 (0.45 to 1.69) | 0.90 (0.47 to 1.71) | - | 0.97 (0.67 to 1.40) | - | 1.44 (1.17 to 1.76)^a^ | 1.14 (0.87 to 1.49) |
| Model 3 | 1.44 (0.44 to 4.68) | 0.84 (0.44 to 1.63) | 0.90 (0.47 to 1.70) | - | 0.98 (0.68 to 1.42) | - | 1.35 (1.10 to 1.66) | 1.12 (0.88 to 1.43) |

Abbreviations: T2D: Type 2 diabetes; ADHD: Attention-deficit/hyperactivity disorder; SES: Socioeconomic status

Note: Model 1 adjusted for sex, age and calendar year at T2D identification; Model 2 additionally adjusted for history of psychiatric disorders, psychotropic medication use and cardiometabolic medication use; Model 3 additionally adjusted for antidiabetic medication use during follow-up. Pooled analysis used a random-effects model to pool the effect sizes from all countries with available results.

^a^Statistically significant after false discovery rate adjustment

# Table S16. Sensitivity analyses: association between ADHD medication use and all-cause mortality following T2D diagnosis across countries

| **Analysis** | **Australia** | **Denmark** | **Netherlands** | **Norway** | **Sweden** | **United Kingdom** | **United States** | **Pooled analysis** |
| --- | --- | --- | --- | --- | --- | --- | --- | --- |
| **Redefine T2D by clinical diagnosis** |  |  |  |  |  |  |  |  |
| Model 1 | - | - | - | - | 0.92 (0.60 to 1.40) | - | - | - |
| Model 2 | - | - | - | - | 0.89 (0.58 to 1.37) | - | - | - |
| Model 3 | - | - | - | - | 0.91 (0.59 to 1.39) | - | - | - |
| **Redefine ADHD by clinical diagnosis** |  |  |  |  |  |  |  |  |
| Model 1 | - | 0.53 (0.21 to 1.34) | 0.65 (0.20 to 2.12) | 1.24 (0.22 to 6.85) | 1.35 (0.92 to 1.97) | - | 1.86 (1.02 to 3.40) | 1.20 (0.79 to 1.83) |
| Model 2 | - | 0.47 (0.18 to 1.26) | 0.66 (0.20 to 2.12) | 0.79 (0.12 to 5.20) | 1.27 (0.87 to 1.87) | - | 1.64 (0.88 to 3.06) | 1.20 (0.89 to 1.61) |
| Model 3 | - | 0.52 (0.20 to 1.35) | 0.64 (0.19 to 2.23) | 0.60 (0.09 to 4.12) | 1.32 (0.90 to 1.94) | - | 1.48 (0.80 to 2.76) | 1.19 (0.88 to 1.60) |
| **Restrict study population to ≤65 years** |  |  |  |  |  |  |  |  |
| Model 1 | 1.34 (0.59 to 3.07) | 0.72 (0.41 to 1.25) | 0.94 (0.49 to 1.80) | 0.91 (0.18 to 4.51) | 1.33 (0.93 to 1.91) | - | 1.97 (1.21 to 3.20) | 1.22 (0.88 to 1.70) |
| Model 2 | 1.46 (0.66 to 3.25) | 0.58 (0.33 to 1.02) | 0.93 (0.48 to 1.80) | 0.77 (0.15 to 4.03) | 1.26 (0.87 to 1.81) | - | 1.85 (1.13 to 3.03) | 1.14 (0.80 to 1.64) |
| Model 3 | 1.47 (0.66 to 3.29) | 0.60 (0.34 to 1.06) | 0.92 (0.47 to 1.77) | 0.67 (0.13 to 3.44) | 1.30 (0.90 to 1.88) | - | 1.68 (1.02 to 2.77) | 1.14 (0.82 to 1.58) |
| **Additionally adjusted for SES** |  |  |  |  |  |  |  |  |
| Model 1 | 1.18 (0.54 to 2.58) | 0.74 (0.47 to 1.17) | 0.91 (0.55 to 1.51) | 0.80 (0.14 to 4.47) | 1.41 (1.00 to 1.98) | - | 1.82 (1.16 to 2.86) | 1.19 (0.87 to 1.61) |
| Model 2 | 1.35 (0.62 to 2.96) | 0.65 (0.41 to 1.04) | 0.91 (0.54 to 1.51) | 0.63 (0.10 to 4.08) | 1.35 (0.95 to 1.90) | - | 1.72 (1.08 to 2.73) | 1.14 (0.83 to 1.56) |
| Model 3 | 1.37 (0.61 to 3.06) | 0.66 (0.41 to 1.06) | 0.99 (0.60 to 1.61) | 0.56 (0.10 to 3.28) | 1.40 (0.99 to 1.97) | - | 1.55 (0.98 to 2.45) | 1.14 (0.85 to 1.53) |

Abbreviations: T2D: Type 2 diabetes; ADHD: Attention-deficit/hyperactivity disorder; SES: Socioeconomic status

Note: Model 1 adjusted for sex, age and calendar year at T2D identification; Model 2 additionally adjusted for history of psychiatric disorders, psychotropic medication use and cardiometabolic medication use; Model 3 additionally adjusted for antidiabetic medication use during follow-up. Pooled analysis used a random-effects model to pool the effect sizes from all countries with available results.

^a^Statistically significant after false discovery rate adjustment

# Figure S1. Study design

Abbreviations: T2D: Type 2 diabetes; T1D: Type 1 diabetes; ADHD: Attention-deficit/hyperactivity disorder; MACE: Major adverse cardiac events

^a^Date of first recorded T2D diagnosis was defined either with a clinical diagnosis of T2D or the prescription of blood glucose-lowering drugs (ATC codes A10B), whichever came first

^b^MACEs included acute myocardial infarction, stroke and cardiovascular death

^c^Psychiatric disorders include: Neurodevelopmental disorders (autism spectrum disorder, intellectual disability), mood and anxiety disorders (anxiety disorders, depressive disorder, bipolar disorder), schizophrenia, personality disorders, eating disorders and substance use disorders

^d^Psychotropic medications include: antiepileptics, antipsychotics, anxiolytics, hypnotics and sedatives, antidepressants, anti-addiction agents

^e^Cardiometabolic medications include: antidiabetic medication, antihypertensive medication, lipid-lowering medication

^f^Earliest of: a) when outcome is MACE: MACE, non-CVD-related death, emigration or end of the study (31^st^ December, 2020); b) when outcome is all-cause mortality: death, emigration or end of the study

# Figure S2. ADHD as time-dependent variable

Abbreviations: T2D: Type 2 diabetes; ADHD: Attention-deficit/hyperactivity disorder

# Figure S3. Example of ADHD medication use as time-dependent variable

Abbreviations: T2D: Type 2 diabetes; ADHD: Attention-deficit/hyperactivity disorder

# References

1. Zoega H, Falster MO, Gillies MB, Litchfield M, Camacho X, Bruno C, et al. The Medicines Intelligence Data Platform: a population-based data resource from New South Wales, Australia. Pharmacoepidemiol Drug Saf. 2024;33:e5887. https://doi.org/10.1002/pds.5887.

2. Laugesen K, Ludvigsson JF, Schmidt M, Gissler M, Valdimarsdottir UA, Lunde A, et al. Nordic health registry-based research: a review of health care systems and key registries. Clin Epidemiol. 2021;13:533–54. https://doi.org/10.2147/CLEP.S314959.

3. Li Y, Zhou Y, Vos M, Bos M, Steen O, Wray NR, et al. Familial co-aggregation and shared heritability between neurodevelopmental problems and cardiometabolic conditions. Nat Ment Health. 2025;3:1545–54. https://doi.org/10.1038/s44220-025-00535-y.

4. Lewis JD, Schninnar R, Bilker WB, Wang X, Strom BL. Validation studies of the health improvement network (THIN) database for pharmacoepidemiology research. Pharmacoepidemiol Drug Saf. 2007;16:393–401. https://doi.org/10.1002/pds.1335.

5. Palchuk MB, London JW, Perez-Rey D, Drebert ZJ, Winer-Jones JP, Thompson CN, et al. A global federated real-world data and analytics platform for research. JAMIA Open. 2023;6:ooad035. https://doi.org/10.1093/jamiaopen/ooad035.

6. Pratt NL, Kerr M, Barratt JD, Kemp-Casey A, Kalisch Ellett LM, Ramsay E, et al. The validity of the Rx-Risk Comorbidity Index using medicines mapped to the Anatomical Therapeutic Chemical (ATC) Classification System. BMJ Open. 2018;8:e021122. https://doi.org/10.1136/bmjopen-2017-021122.
